# Circulating miR-320b Contributes to CD4+ T-Cell Proliferation in Systemic Lupus Erythematosus via MAP3K1

**DOI:** 10.1155/2023/6696967

**Published:** 2023-10-26

**Authors:** Zutong Li, Rou Wang, Dandan Wang, Shujie Zhang, Hua Song, Shuai Ding, Yantong Zhu, Xin Wen, Hui Li, Hongwei Chen, Shanshan Liu, Lingyun Sun

**Affiliations:** ^1^Department of Rheumatology and Immunology, Nanjing Drum Tower Hospital, Affiliated Hospital of Medical School, Nanjing University, Nanjing, China; ^2^Department of Rheumatology and Immunology, Nanjing Drum Tower Hospital Clinical College of Nanjing University of Chinese Medicine, Nanjing, China; ^3^MOE Key Laboratory of Model Animals for Disease Study, Model Animal Research Center, Medical School of Nanjing University, Nanjing, China; ^4^Department of Rheumatology, Affiliated Hospital of Shandong University of Traditional Chinese Medicine, Jinan, China

## Abstract

Systemic lupus erythematosus (SLE) is a chronic autoimmune disease characterized by the production of autoantibodies and tissue inflammation. Mesenchymal stem cells (MSCs) have emerged as a promising candidate therapy for SLE owing to the immunomodulatory and regenerative properties. Circulating miRNAs are small, single-stranded noncoding RNAs in a variety of body fluids that regulate numerous immunologic and inflammatory pathways. Recent studies have revealed many differentially expressed circulating miRNAs in autoimmune diseases including SLE. However, the role of circulating miRNAs in SLE has not been extensively studied. Here, we performed small RNA sequencing analysis to compare the circulating miRNA profiles of SLE patients before and after MSC transplantation (MSCT), and identified a significant decrease of circulating miR-320b level during MSCT. Importantly, we found that the expression of circulating miR-320b and its target gene MAP3K1 was closely associated with SLE disease activity. The *in vitro* experiments showed that decreased MAP3K1 level in SLE peripheral blood mononuclear cells (PBMCs) was involved in CD4+ T-cell proliferation. In MRL/*lpr* mice, miR-320b overexpression aggravated symptoms of SLE, while miR-320b inhibition could promote disease remission. Besides, MSCs regulate miR-320b/MAP3K1 expression both *in vitro* and *in vivo*. Our results suggested that circulating miR-320b and MAP3K1 may be involved in CD4+ T-cell proliferation in SLE. This trial is registered with NCT01741857.

## 1. Background

Systemic lupus erythematosus (SLE) is a chronic autoimmune disease characterized by dysregulation of T and B lymphocytes and loss of immune tolerance to self-antigens. CD4+ T-helper cells play crucial roles in orchestrating immune responses by providing costimulatory signals and cytokines, which contribute to autoantibody production and organ damage [1]. However, the pathogenesis of SLE is incompletely understood. Recently, circulating miRNAs have been shown to play an important role in SLE.

Circulating miRNAs are small, single-stranded noncoding RNAs in a variety of body fluids that regulate numerous immunologic and inflammatory pathways by inhibiting mRNA translation or promoting mRNA degradation [2, 3]. SLE patients have unique miRNA signatures in peripheral blood cells, plasma, and body fluids. Several circulating miRNAs, such as miR-21, miR-155, miR-125a-3p, and miR-146a, have been proposed as diagnostic and prognostic biomarkers in SLE patients [4–7]. In lupus, CD4+ T cells are critical drivers of the antibody response and tissue injury. The dysregulated expansion of T follicular helper (Tfh) cells and Th17 cells contributed to the pathogenesis of SLE [8]. Recent studies have shown that circulating miRNAs can modulate the function and phenotype of T-cell subsets. Circulating exosomal miR-17 from rheumatoid arthritis patients inhibited the induction of regulatory T cells (Treg) via suppressing TGFBR II expression *in vitro* [9]. Furthermore, exosomal miR-142 was found to regulate CD4+ T-cell immunometabolic dysfunction and exacerbate cardiac injury in mice with experimental autoimmune myocarditis [10]. Circulating miR-221/222 reduced the number of peripheral CD4+ T cells by inhibiting CD4 expression in colorectal cancer [11]. Besides, let-7i and miR-208b were found to regulate Treg expansion [12, 13]. Despite these studies in other diseases, the function and molecular mechanisms of circulating miRNAs in lupus CD4+ T cells are not well understood.

Mesenchymal stem cells (MSCs) are multipotent nonhematopoietic progenitors that can modulate various immune cells such as T cells, B cells, dendritic cells, and macrophages through cell–cell contact, cytokine secretion, and other mechanisms [14–18]. Due to their low immunogenicity and immunomodulatory properties, MSC transplantation (MSCT) has been considered as a potential therapy for autoimmune diseases. Clinical studies have demonstrated the safety and efficacy of allogeneic umbilical cord-derived MSCs (UC-MSCs) in SLE patients refractory or intolerant to conventional therapies [19–22]. To date, there have been no reports about the profiles of circulating miRNAs during MSCT, and the roles of circulating miRNAs in mediating the immunomodulatory function of MSCT in SLE remain unclear.

In this study, we investigated the circulating miRNA profile in SLE patients pre- and post-MSCT, and identified the potential diagnostic value of circulating miR-320b and its target gene MAP3K1. We also explored the effects of MAP3K1 on CD4+ T-cell proliferation *in vitro* by downregulating MAP3K1 expression in peripheral blood mononuclear cells (PBMCs). Furthermore, we evaluated the *in vivo* role of miR-320b by inhibiting its expression in the plasma of MRL/*lpr* mice and observing the amelioration of SLE symptoms. These results have important implications for the diagnosis and treatment of SLE.

## 2. Methods

### 2.1. Subjects

From May 2018 to December 2022, 40 SLE patients were enrolled in an allogeneic MSCT trial carried out at the Department of Rheumatology and Immunology, Nanjing Drum Tower Hospital, Affiliated Hospital of Medical School, Nanjing University. All enrolled patients fulfilled at least four of 11 American College of Rheumatology criteria for the classification of SLE. Plasma samples (*n* = 3) were analyzed by miRNA sequencing, and plasma (*n* = 32) and PBMCs (*n* = 7) samples were used for validation. Besides, blood from 46 SLE patients and 30 sex- and age-matched healthy controls was obtained from this hospital between May 2019 and May 2020. Written informed consent was obtained from all patients and healthy donors that provided blood samples 10 mL or more specifically for the study. When only residual blood was used, written informed consent was waived. The disease activity was evaluated according to SLE Disease Activity Index (SLEDAI)-2K score [23]. The level of hemoglobin, serum albumin, serum 25-hydroxyvitamin D (25-(OH)D), and other clinical data of these enrolled patients were collected and analyzed. In this research, responders were patients with low disease activity states during follow-up period, with the criteria of a SLEDAI score ≤3 on antimalarials, or alternatively SLEDAI ≤4, physician global assessment (PGA) ≤1 with glucocorticoids (GC) ≤7.5 mg of prednisone and well-tolerated immunosuppressive agents. Patients who did not meet either of the above criteria were classified as nonresponders [24].

### 2.2. Preparation and Infusion of UC-MSCs

For clinical use, UC-MSCs were prepared by the Stem Cell Center of Jiangsu Province (Beike Biotechnology) as previously described [25]. In this study, 40 patients underwent MSCT. One million cells per kilogram of body weight were administered by intravenous infusion. The study protocol was registered on ClinicalTrials.gov (identifier: NCT01741857). The demographics and clinical characteristics of the patients were summarized in *Supplementary 1*.

### 2.3. miRNA Library Construction, Sequencing, and Analysis

Total RNA in plasma was extracted using TRIzol Reagent (Invitrogen, Carlsbad, CA, USA). The library construction, sequencing, and differential expression analysis were performed by BGI Genomics Co., Ltd. (Shenzhen, China) on the BGISEQ-500 platform. *p*-value < 0.01 and |log2FoldChange| > 1 were set as the threshold. The target genes were predicted by multiMiR (including miRecords, miRTarBase, TarBase, DIANA-microT, ElMMo, MicroCosm, miRanda, miRDB, PicTar, PITA, and TargetScan microRNA-target databases). To annotate gene functions, target genes were aligned against the Kyoto Encyclopedia of Genes and Genomes (KEGG) and Gene Ontology (GO) databases. GO and KEGG enrichment analyses were performed using ClusterProfiler with R.

### 2.4. Quantitative Real-Time PCR

For plasma samples, small RNA was extracted using the miRNeasy Serum/Plasma Kit according to the manufacturer's instructions, and 5.6 × 10^8^ copies of a *Caenorhabditis elegans* miRNA (cel-39) were added to each sample as a spike-in control. The qRT-PCR was performed with cDNA synthesized with miScript II RT Kit, SYBR Green Master Mix (all purchased from QIAGEN, Hilden, Germany) and specific miRNA primers (GenScript, Nanjing, China). The relative expression of miRNAs was determined and normalized to the expression of cel-39 and calculated using the 2^−*ΔΔCt*^ method.

Total RNAs from cultured cells or PBMCs were extracted using TRIzol Reagent and reverse-transcribed into cDNA using HiScript II Q RT SuperMix (Vazyme Biotech, Nanjing, China). For miRNA detection, cDNA was synthesized with *Escherichia coli* Poly(A) Polymerase (NEB, USA) and HiScript II Q Select RT SuperMix (Vazyme Biotech, Nanjing, China). The relative gene quantification was normalized to GAPDH or U6. All primers were listed in *Supplementary 1*.

### 2.5. Western Blot Analysis

We used antibodies recognizing human MAP3K1 (1 : 600, Proteintech, Wuhan, China) and *α*-tublin (1 : 1000, Sigma, USA) to examine the concentrations of proteins in HEK293T lysates. Images were captured and analyzed by the Tanon-5200 Chemiluminescent Imaging System.

### 2.6. Dual-Luciferase Assay

The sequence of MAP3K1 3′-UTR and 3′-UTR-mutant regions was synthesized and cloned into the pmirGLO Dual-Luciferase miRNA vector (Promega, Madison, USA) by GenScript Company (Nanjing, China). HEK293T cells were cotransfected with the luciferase reporter constructed (500 ng) together with miRNA mimics or negative control (NC, 40 nM) using Lipofectamine 3000. After 48 hr, the cells were washed and lysed in the passive lysis buffer (Promega, Madison, USA). Firefly luciferase (f-luc) and renilla luciferase (r-luc) activities were detected by a dual-luciferase reporter assay system (Promega, Madison, USA). Relative reporter activity was normalized to the r-luc activity.

### 2.7. Lentivirus Infection

The specific siRNA of MAP3K1 and scrambled siRNA control were designed and synthesized by GenePharma (Shanghai, China). The shMAP3K1 plasmid was constructed and synthesized using the pSIH vector by GenScript Company (Nanjing, China). Lentivirus was packaged by cotransfection of psPAX2, pMD2.G, and shMAP3K1 plasmids into HEK293T cells. PBMCs were cultured in a 96-well plate in the presence of polybrene (1 : 1,000, Sigma-Aldrich), with the addition of 30 *μ*L lentivirus for 3 days.

### 2.8. UC-MSC and PBMC Coculture

UC-MSCs were obtained, isolated, and preserved as previously described [18, 26]. The cells were cultured with Dulbecco's Modified Eagle Medium (DMEM)/F12 supplemented with 10% fetal bovine serum (FBS) and 100 U/mL penicillin/streptomycin (Gibco, NY, USA). In the coculture experiment, UC-MSCs (1.0 × 10^4^) were seeded in 48-well culture plate overnight. PBMCs (4 × 10^5^ cells/mL) from healthy controls were labeled with eFluor™ 450 (eBioscience, CA, USA) according to the manufacturer's protocol and cocultured with UC-MSCs directly, with the addition of soluble anti-CD3 and anti-CD28 (1 *μ*g/mL, all purchased from Thermo Fisher Scientific, MA, USA) monoclonal antibodies. In RNase group, the cells were also treated with RNase A (100 *μ*g/mL, NEB, USA). After 4 days, the cells were harvested for the following detection by cytometry.

### 2.9. Cell Proliferation Assay

The procedure for lentivirus infection and coculture has been described above. Then, the cells were harvested and stained with phycoerythrin (PE)-CF594-conjugated antihuman CD4, antihuman CD8 PerCP/Cy5.5, and eFluor™ 780 (eBioscience, CA, USA). In mice, single-cell suspensions of spleens were prepared and stained with antimouse CD4 FITC, PE-Cy7-conjugated antimouse Ki67 (eBioscience, CA, USA), eFluor™ 780, antimouse CD3a BUV395, and antimouse CD8a BV510 (BD Pharmingen, USA). Data were collected by BD LSRFortessa™ Flow Cytometer and analyzed by FlowJo software.

### 2.10. Animals

Fourteen female MRL/*lpr* mice were purchased from SLRC Experimental Animals Co., Ltd. (Shanghai, China) and maintained in a controlled environment (20 ± 2°C, 12-hr/12-hr light/dark cycle) under specific pathogen-free conditions at the Nanjing Drum Tower Hospital, Affiliated Hospital of Medical School, Nanjing University. All animal studies were approved by Animal Care and Use Committee of the hospital. All procedures were performed in accordance with the guidelines of the hospital.

Sixteen-week-old MRL/*lpr* mice were divided into NC (*n* = 5), miR-320b agomir (*n* = 4), and miR-320b antagomir (*n* = 5) groups. A total of 20 nmol miR-320b agomirs, antagomirs, or NC were injected intravenously every 6 days for four times. All mice were sacrificed 19 days after the first administration. Agomirs, antagomirs, and NC were purchased from RiboBio Co., Ltd. (Guangzhou, China). Urine and blood samples were collected every week until the end of the experiment. The concentrations of urinary protein were measured using Bradford protein quantitation assay (KeyGen, Nanjing, China). Cytokine concentrations were measured by ELISA (MultiSciences Biotech, Nanjing, China) according to the manufacturer's instructions.

### 2.11. Renal Histopathologic Analysis

Murine kidneys were fixed in 4% paraformaldehyde for 24 hr, embedded in paraffin, and sectioned at 3 *μ*m. The sections were stained with hematoxylin and eosin (H&E), periodic acid–Schiff (PAS), and Masson's trichrome stain, respectively. The histological scores for glomerular, interstitial, and perivascular lesions were measured as previously described [27].

### 2.12. Immunofluorescence

Slides were washed with phosphate-buffered saline (PBS) and fixed with 4% paraformaldehyde for 30 min and measured with 0.2% Triton-X 100 for 10 min. Then, they were blocked with 5% bovine serum albumin and incubated with Alexa Fluor 555-antimouse IgG (H + L) (Cell Signaling Technology, MA, USA) for 1 hr at room temperature. For the staining of complement 3 (C3), frozen sections were incubated with rabbit antimouse C3 (Abcam, MA, USA) followed by the staining of secondary Alexa Fluor 488-antirabbit IgG (H + L) (Proteintech, Wuhan, China). After three PBS washes, the slides were counterstained with DAPI for 1 min and then observed under the Confocal Laser Scanning Microscope FV3000 (Olympus, Tokyo, Japan).

### 2.13. Statistical Analysis

Statistical analysis was performed with Prism 8 (GraphPad). The data are presented as the mean ±SEM. Differences between pre- and post-MSCT samples were determined by paired Student's *t*-test and the variance was normally distributed. Unpaired Student's *t*-test was used to detect the differences between the two groups. Pearson correlation analysis was used to evaluate correlations, and *p*-values < 0.05 were considered as statistically significant.

## 3. Results

### 3.1. Circulating miR-320b Is Decreased after MSCT in SLE Patients and Associated with Disease Activity

**Figure 1:**
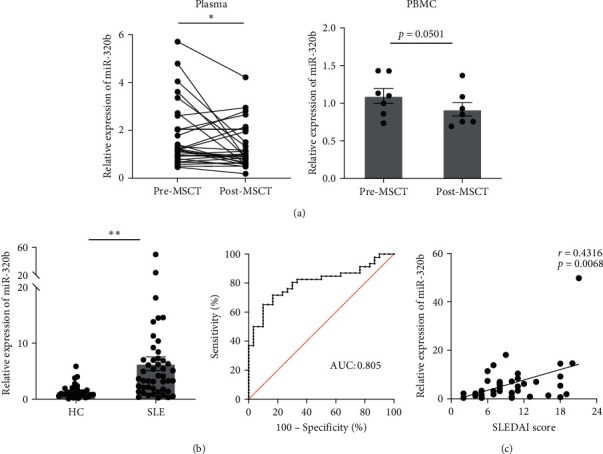
Circulating miR-320b is decreased after MSCT in SLE patients and associated with disease activity. (a) miR-320b expression in plasma and PBMCs of SLE patients before and after receiving MSCT. Plasma, *n* = 32; PBMC, *n* = 7. (b) Expression levels and ROC curve of circulating miR-320b in HC and SLE groups. HC, *n* = 30; SLE, *n* = 46. (c) Association of circulating miR-320b expression with the SLEDAI score of SLE patients. HC, healthy control; MSCT, mesenchymal stem cell transplantation; ROC, receiver operating characteristic; SLEDAI, systemic lupus erythematosus disease activity index.  ^*∗*^*p* < 0.05,  ^*∗*^ ^*∗*^*p* < 0.01.

We performed miRNA sequencing in plasma samples derived from three SLE patients before and after MSCT and identified 104 upregulated and 35 downregulated miRNAs (*Supplementary 1, 2, and 3*). We validated the expression of specific miRNAs in the plasma of an additional 30 SLE patients based on the screening results, and found that the level of circulating miR-320b was significantly decreased in post-MSCT samples compared to pre-MSCT samples (*p* < 0.05). Similarly, the miR-320b level also decreased in PBMCs of post-MSCT group (*p*=0.0501) (Figure 1(a)).

Moreover, miR-320b expression was significantly elevated in the plasma of SLE patients compared with healthy controls. The area under the curve (AUC) for miR-320b in distinguishing SLE patients from healthy controls was 0.805 (Figure 1(b)). Our data showed that miR-320b level was positively correlated with the SLEDAI score (Figure 1(c)), indicating that circulating miR-320b may be involved in the pathogenesis of SLE.

### 3.2. MAP3K1 Is a Direct Target of miR-320b

**Figure 2:**
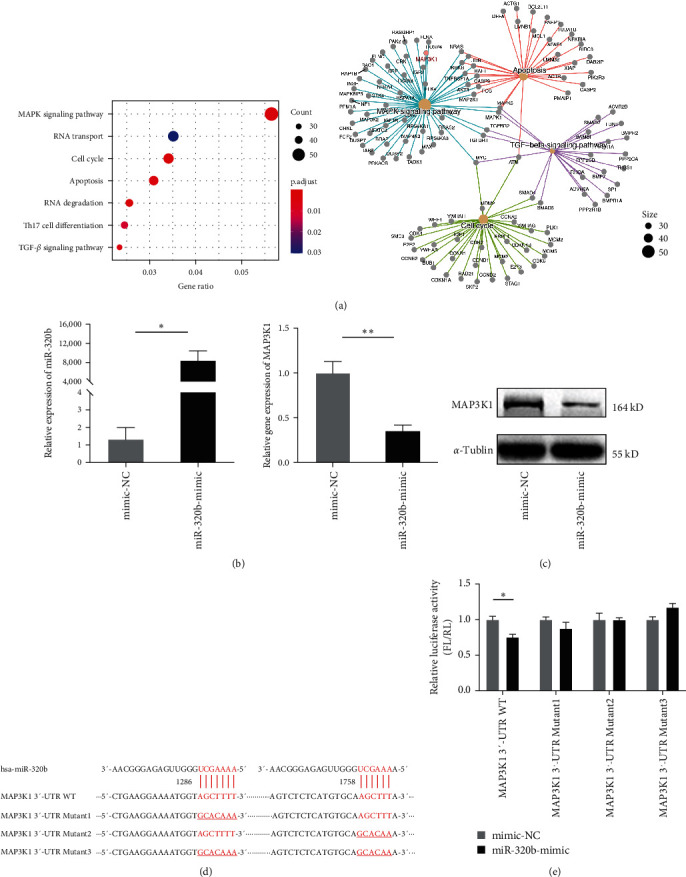
MAP3K1 is a target of miR-320b. (a) The KEGG pathway analysis of predicted target genes of miR-320b. (b) HEK293T was transfected with mimics of miR-320b for 48 hr. MAP3K1 mRNA abundance was measured using qRT-PCR. (c) Protein levels were assessed using Western blot. (d) Sequence analysis of human miR-320b and MAP3K1 mRNA 3′-UTR. The predicted binding regions are highlighted in red. (e) Luciferase activity assays were performed to assess whether the regulatory effect of hsa-miR-320b requires the predicted binding sites in the MAP3K1 mRNA 3′-UTR in HEK293T cells. For miR-320b, the respective binding sites were individually mutated in pMIR1286 (Mutant1), pMIR1758 (Mutant2), and both two sites (Mutant3). *n* = 3 per group.  ^*∗*^*p* < 0.05,  ^*∗*^ ^*∗*^*p* < 0.01.

To identify candidate miR-320b target genes, we found that the target genes were mainly enriched in mitogen-activated protein kinase (MAPK) signaling pathways by using TargetScan (http://www.targetscan.org) and KEGG pathway analysis (Figure 2(a)). Among the predicted targets, MAP3K1 mRNA and protein levels were significantly lower in cells transfected with miRNA mimics (Figures 2(b) and 2(c)). Bioinformatic analysis predicted two highly conserved binding sites (1,286–1,292 and 1,758–1,763) in the MAP3K1 mRNA 3′-UTR (Figure 2(d)). Luciferase reporter assays showed that miR-320b directly regulated MAP3K1 expression by binding to two conserved sites of 3′-UTR of the mRNA (Figure 2(e)).

### 3.3. MAP3K1 Level Is Decreased in SLE Patients and Involved in CD4+ T-Cell Proliferation

**Figure 3:**
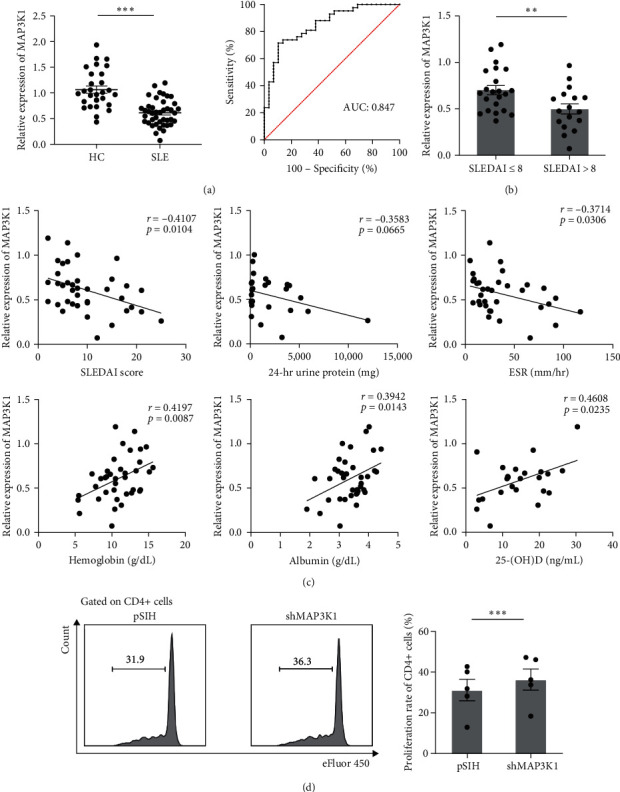
Decreased MAP3K1 level in SLE PBMCs is involved in CD4+ T-cell proliferation. (a) The MAP3K1 expression in PBMCs and ROC curve of HC and SLE groups. HC, *n* = 29; SLE, *n* = 42. (b) The MAP3K1 expression was compared between SLE patients with higher (*n* = 17) and lower disease activities (*n* = 22). (c) Correlations between MAP3K1 expression with SLEDAI score, 24-hr urine protein, ESR, hemoglobin, albumin, and 25-(OH) D levels in SLE patients. (d) PBMCs were infected with shMAP3K1 lentivirus and cultured for 3 days. The proliferation rate of CD4+ T cells was measured byflow cytometry. *n* = 5. ESR, erythrocyte sedimentation rate; HC, healthy control; PBMC, peripheral blood mononuclear cell; ROC, receiver operating characteristic; SLEDAI, systemic lupus erythematosus disease activity index. ^*∗*^ ^*∗*^*p* < 0.01,  ^*∗*^ ^*∗*^ ^*∗*^*p* < 0.001.

Next, we detected MAP3K1 expression in PBMCs from healthy controls and SLE patients and found that MAP3K1 was significantly decreased in SLE patients. We then evaluated the diagnostic ability of MAP3K1 in SLE using ROC and obtain the AUC on MAP3K1 with a value of 0.847 (Figure 3(a)). We also observed a significant difference in MAP3K1 mRNA levels in PBMCs between SLE patients with higher disease activity (SLEDAI > 8) and those with lower activity (SLEDAI ≤ 8) (Figure 3(b)). Moreover, further analysis showed that MAP3K1 expression was negatively correlated with SLEDAI and erythrocyte sedimentation rate (ESR) levels (*p* < 0.05), and positively correlated with hemoglobin, serum albumin, and serum 25-(OH)D levels (*p* < 0.05). Besides, MAP3K1 expression showed a negative tendency with the level of 24-hr urine protein (*p*=0.0665) (Figure 3(c)).

Previous studies have shown aberrant T-cell proliferation in SLE patients and lupus mice [28]. We wondered whether MAP3K1 could play a role in regulating immune cell proliferation. Then, we performed MAP3K1 expression knockdown in HC PBMCs by shMAP3K1 lentivirus and found elevated proliferation rate of CD4+ T cells (*Supplementary 1* and Figure 3(d)). These data revealed that decreased MAP3K1 levels in SLE PBMCs may be involved in CD4+ T-cell proliferation.

### 3.4. miR-320b Blockade Alleviates Symptoms of SLE and Inhibits Proliferation of CD4+ T Cells in MRL/*lpr* Mice

**Figure 4:**
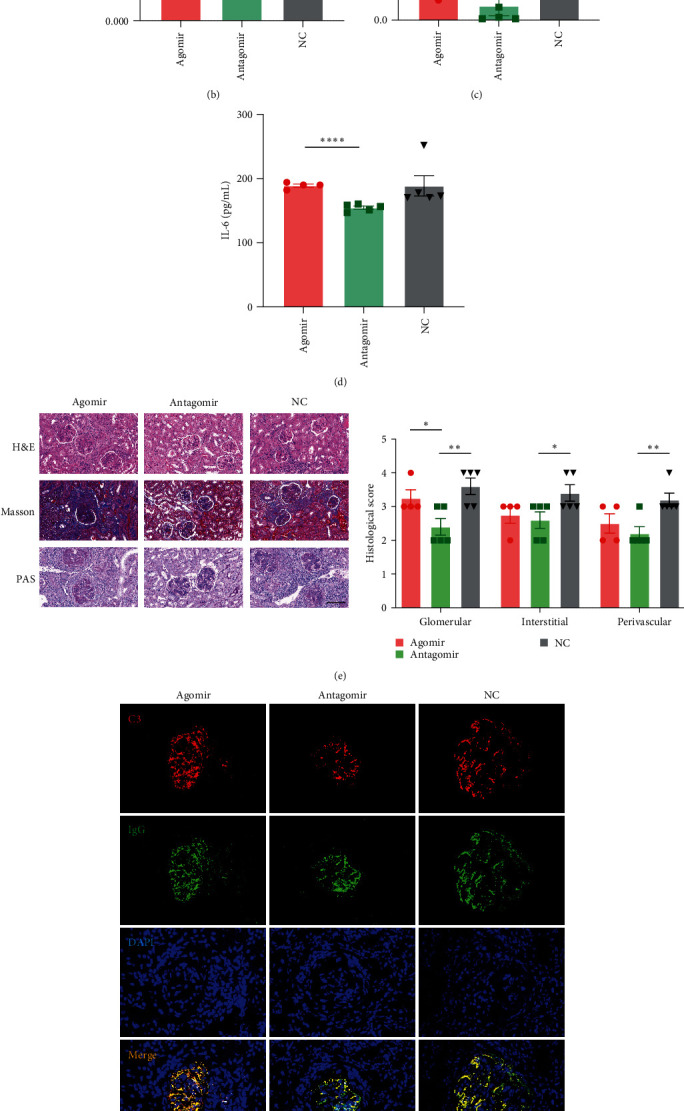
miR-320b blockade alleviates symptoms of SLE and inhibits proliferation of CD4+ T cells in MRL/*lpr* mice. (a) The design diagram of the *in vivo* experiment. (b–d) The spleen/body weight index (b), urine protein levels (c), and plasma levels of IL-6 (d) in agomir, antagomir, and NC group. (e) Representative images and histological score of kidney lesions staining with H&E, Masson, and PAS. Scale bar, 100 *μ*m. (f) Representative images of C3 and IgG immunostaining in glomeruli. Scale bar, 50 *μ*m. (g) The rates of splenic Ki67+ CD4+ T cells were detected by flow cytometry. (h) The mRNA levels of MAP3K1 of spleens in three groups. agomir, *n* = 4; antagomir, *n* = 5; NC, *n* = 5. H&E, hematoxylin and eosin; NC, negative control; PAS, periodic acid–Schiff.  ^*∗*^*p* < 0.05,  ^*∗*^ ^*∗*^*p* < 0.01,  ^*∗*^ ^*∗*^ ^*∗*^ ^*∗*^*p* < 0.0001.

The role of miR-320b in MSCT of lupus mice was investigated by injecting agomirs, antagomirs, or NC in MRL/*lpr* mice (Figure 4(a)). The results showed that the spleen/body mass index (Figure 4(b)), urine protein level (Figure 4(c)), and plasma IL-6 level (Figure 4(d)) were significantly decreased in the miR-320b antagomir group compared with those in miR-320b agomir and NC groups. Renal impairments were also ameliorated in miR-320b antagomir group, as shown by reduced inflammatory cell infiltration, tubular atrophy, fibrosis (Figure 4(e)), and less C3 and IgG deposition in the peripheral capillary loops (Figure 4(f)). These *in vivo* findings demonstrated that miR-320b blockade exerted therapeutic effects in MRL/*lpr* mice.

We also detected the proliferation of T cells in different organs and found that the proliferation rate of splenic CD4+ T cells in the miR-320b antagomir group was significantly lower than that in the NC group (Figure 4(g)). Furthermore, the injection of miR-320b antagomirs significantly increased the mRNA levels of MAP3K1 in the spleens of these MRL/*lpr* mice (Figure 4(h)). Thus, miR-320b blockade inhibited the proliferation of splenic CD4+ T cells in MRL/*lpr* mice. These results suggest that miR-320b may play a crucial role in lupus mice by regulating MAP3K1 expression.

### 3.5. MSCs Regulate miR-320b/MAP3K1 Expression Both *In Vitro* and *In Vivo*

**Figure 5:**
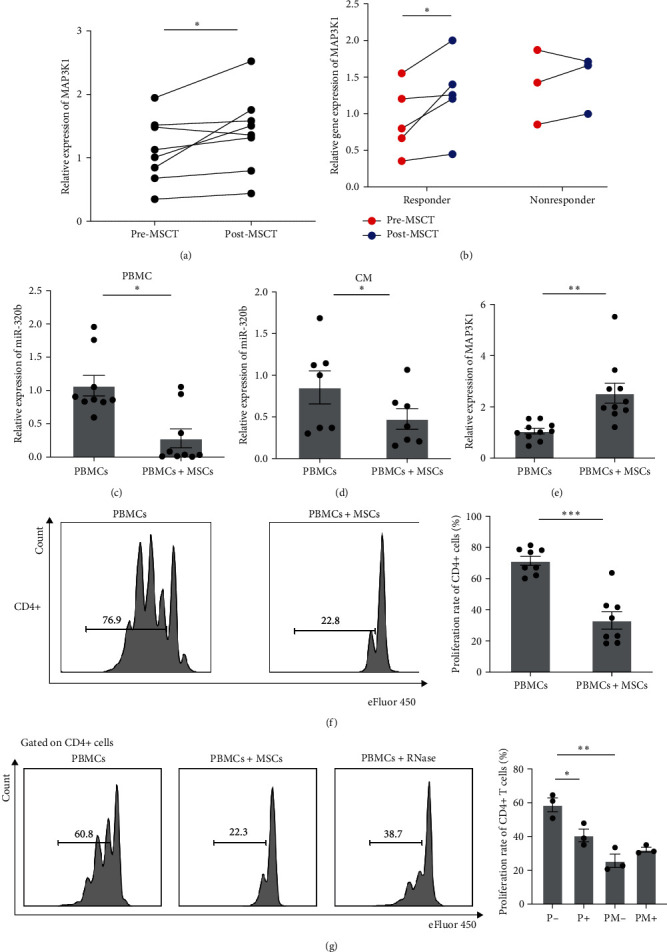
MSCs regulate miR-320b/MAP3K1 expression both *in vitro* and *in vivo*. (a) PBMCs levels of MAP3K1 in SLE patients receiving MSCT. *n* = 8. (b) The changes in MAP3K1 expression in responders (*n* = 5) and nonresponders (*n* = 3) during MSCT. (c–f) PBMCs from HC were isolated, stimulated, and cocultured with or without MSCs for 4 days. The expression levels of miR-320b in PBMCs (c) and coculture medium (d) and MAP3K1 in PBMCs (e) were compared between two groups. The proliferation rate of CD4+ T cells was measured by flow cytometry (f). (c) *n* = 9; (d) *n* = 7; (e) *n* = 10. (g) PBMCs from HC were isolated, stimulated, and cultured in the presence of MSCs or RNase for 4 days. The proliferation rate of CD4+ T cells was measured by flow cytometry. *n* = 3. MSCT, mesenchymal stem cell transplantation; PBMC, peripheral blood mononuclear cell. ^*∗*^*p* < 0.05,  ^*∗*^ ^*∗*^*p* < 0.01,  ^*∗*^ ^*∗*^ ^*∗*^*p* < 0.001.

We compared MAP3K1 expression levels in patient PBMCs before and after MSCT and found that MAP3K1 expression was increased after MSCT (Figure 5(a)). Additionally, we examined MAP3K1 levels in PBMCs from MSCT patients who had been followed-up for 6 months. The data showed that MAP3K1 expression levels increased in five patients who had the clinical response after MSCT, whereas the level remained unchanged in three patients who had no response (Figure 5(b)).

To explore whether MSCs can regulate miR-320b/MAP3K1 expression *in vitro*, we conducted coculture experiments using PBMCs and MSCs. We found that miR-320b level was both significantly decreased in PBMCs (Figure 5(c)) and coculture medium (Figure 5(d)), and MAP3K1 mRNA level was increased in PBMCs (Figure 5(e)). Besides, the cell proliferation assay showed that MSCs inhibited CD4+ T-cell proliferation (Figure 5(f)).

Next, we used RNase to abrogate the expression of miRNAs in the coculture supernatant. Compared with PBMCs cocultured with MSCs without RNase, the proliferation rates of PBMCs and CD4+ T cells were significantly decreased in PBMCs with RNase (Figure 5(g)). These results suggested that circulating miRNAs, including miR-320b, were involved in the inhibitory effects of T-cell proliferation.

## 4. Discussion

SLE is a complex autoimmune disease characterized by the production of autoantibodies and the involvement of multiple organs. MSCT has been demonstrated as a safe and effective therapy for SLE. Although several circulating miRNAs have been characterized as potential diagnostic markers or therapeutic targets for SLE, whether circulating miRNAs play functional roles in the pathogenesis of SLE remains unclear. In our study, we found that MSCs regulate circulating miR-320b and miR-320b/MAP3K1 expression to restrain CD4+ T-cell proliferation in SLE and lupus mice. These results suggested that miR-320b may be a potential diagnostic markers and target for the treatment of SLE.

Here, we performed small RNA sequencing analysis to identify the differentially expressed circulating miRNAs during MSCT. Our data showed that miR-320b was significantly decreased in the plasma of SLE patients after MSCT. Importantly, miR-320b expression was associated with SLEDAI and other clinical parameters. In SLE patients, circulating miRNAs were reported to be related to disease activities [7, 29–31] and associated with organ involvement, including lupus nephritis and thrombopenia [32–37]. These miRNAs have been characterized as potential diagnostic markers or therapeutic targets. Therefore, our results indicated that miR-320b may serve as new potential diagnostic biomarker for SLE.

In recent studies, miR-320b has been reported to suppress cell proliferation, migration, and invasion in various cancers [38]. However, there are few studies on miR-320b in immune diseases, including SLE. To further explore the biological function of miR-320b in SLE, we screened out and analyzed the expression of miR-320b target gene MAP3K1. MAP3K1 is a member of the MAP3K superfamily that controls the MAPKK-MAPK signaling cascades and regulates various aspects of cell biology, most notably cell proliferation. Lupus T cells exhibit autoreactive and activated inflammatory phenotype, which contribute to the pathogenesis of SLE [28]. Herein, we used the miRNA agomir/antagomir *in vivo* and found that antagomir treatment alleviates the symptoms of LN and inhibits the proliferation of CD4+ T cells in MRL/*lpr* mice. The knockdown of MAP3K1 promoted the proliferation of T cells *in vitro*. This inhibitory effect strengthened the potential clinical significance of miR-320b and downstream target MAP3K1 in SLE. One study has similarly shown that knockout of MAP3K1 can promote cell proliferation during retinal development [39]. They suggested that MAP3K1 suppressed the expression of cyclin D1 and CDK4/6, thereby inhibiting E2F activity for gene expression and DNA replication G1 to S-phase transition in dividing cells. Despite these data, MAP3K1 was also reported involved in CD40 ligand-induced cyclin D2 expression and proliferation in B cells [40]. MAP3K1 may negatively regulate the CDK-RB-E2F pathway in multiple cell types. Our findings suggested that MAP3K1 also represented potential diagnostic biomarkers and miR-320b/MAP3K1 may be involved in the CD4+ T-cell proliferation in SLE. However, the regulatory effects and molecular mechanisms need further investigation.

Previous studies have shown that MSCs have an inhibitory effect on the proliferation and cytokine secretion of T cells. Cytokines like transforming growth factor-*β* (TGF-*β*), nitric oxide (NO), prostaglandin E2 (PGE2), and indoleamine-2,3-dioxygenase (IDO) were involved in the MSC-mediated T-cell suppression [14]. Our group also reported that MSCs suppress the proliferation of T cells through a CD8+ T cell/IFN-*γ*/IDO axis [15]. In this study, we found that MSCs regulated miR-320b/MAP3K1 expression both *in vitro* and *in vivo* and were possibly involved in the inhibitory effects of T-cell proliferation.

Recent researches suggest that MSCs can exhibit therapeutic effects through regulation of miRNAs in autoimmune diseases. In SLE, MSCT rescued bone marrow-derived MSCs function in MRL/*lpr* mice by transferring Fas and downstream miR-29b/Dnmt1/Notch epigenetic cascade [41]. Despite these findings, the molecular mechanisms of MSCs in the regulation of circulating miRNAs remain elucidated. Circulating miRNAs are secreted into the extracellular space by different cell types, in the form of lipid or lipoprotein complex, such as microvesicles and exosomes [42]. They are delivered to circulating cells or other tissue cells [43, 44] and play a role in cell–cell communication [45]. In recent studies, miRNAs were mainly secreted or transferred from MSC-derived extracellular vesicles (EVs) to regulate the biological functions of cells. MSCs engineered to overexpress miRNA-let7c attenuated kidney damage and fibrosis in mice with unilateral ureteral obstruction through miRNA transfer [46]. In septic mice, IL-1*β*-pretreated MSCs transferred exosomal miR-146a to macrophages, leading to M2 polarization and improved survival rate [47]. However, MSC-derived EVs did not include miR-320b (data not shown), and miR-320b expression in plasma and PBMCs was both decreased in patients receiving MSCT. There are some possible speculations regarding the changes of miR-320b expression during MSCT. Some MSC-derived miRNAs may regulate DNA methyltransferase expression, such as DNMT1, DNMT3a, etc. [48] We suggest that some miRNAs from MSCs may regulate circulating and cellular miR-320b expression through DNA methyltransferase in PBMCs. MSC-derived lncRNAs may target miR-320b and downregulate its expression, thereby regulating MAP3K1 expression in PBMCs [49]. Therefore, further investigations are needed to clarify the underlying mechanisms of MSCs-regulated circulating miRNAs in the treatment of SLE.

There are still several limitations in this study. First of all, the molecular mechanisms of miR-320b/MAP3K1 axis in the pathogenesis and treatment of SLE remain unclear. Besides, although many miRNAs have been characterized as diagnostic and prognostic biomarkers for SLE, further research is urgently needed regarding their clinical and therapeutic effects. To date, no clinical research has been conducted on miRNA therapy for the treatment of SLE. This may be due to the insufficient safety of this therapy and the difficulty in ensuring targeted delivery of miRNA. Thus, further experiments are needed to clarify the functional roles and underlying mechanisms of miR-320b/MAP3K1 in SLE.

## 5. Conclusion

In conclusion, our findings suggested that circulating miR-320b and its target gene MAP3K1 represent potential diagnostic biomarkers and may be involved in the CD4+ T-cell proliferation in SLE. These results provide new insights into the pathogenesis of SLE and suggest potential targets for the treatment of this disease.

## Data Availability

The data used to support the findings of this study are included within the supplementary files.

## Abbreviations

3′-UTR: 3′-Untranslated regions
Treg: Regulatory T cell
UC-MSCs: Umbilical cord-derived mesenchymal stem cells
MAPK: Mitogen-activated protein kinase
ESR: Erythrocyte sedimentation rate
25-(OH)D: 25-hydroxyvitamin D
AUC: Area under the curve
C: Complement
EV: Extracellular vesicles
FITC: Fluorescein isothiocyanate
GO: Gene Ontology
HC: Healthy control
H&E: Hematoxylin and eosin
IDO: Indoleamine-2,3-dioxygenase
KEGG: Kyoto Encyclopedia of Genes and Genomes
miRNA: microRNA
MSCs: Mesenchymal stem cells
MSCT: Mesenchymal stem cell transplantation
NC: Negative control
NO: Nitric oxide
NPSLE: Neuropsychiatric systemic lupus erythematosus
PAS: Periodic acid–Schiff
PBMCs: Peripheral blood mononuclear cells
PE: Phycoerythrin
PGE2: Prostaglandin E2
ROC: Receiver operating characteristic
SLE: Systemic lupus erythematosus
SLEDAI: Systemic lupus erythematosus disease activity index
TGF-*β*: Transforming growth factor-*β*
WT: Wild type.

## Supplementary Materials

Supplementary 1 Table S1: demographics and characteristics of SLE patients in miRNA sequencing. Table S2: demographics and characteristics of SLE patients for validation of miRNA candidates. Table S3: primers for reverse transcription and real-time polymerase chain reaction. Figure S1: the heatmap (a) and volcano plot (b) of differentially expressed miRNAs in SLE patients before and after receiving MSCT. Figure S2: the infection of PBMCs with shMAP3K1 lentivirus.

Supplementary 2 Total miRNA expressions detected in the small RNA sequencing analysis of pre- and post-MSCT group.

Supplementary 3 The differentially expressed miRNAs between pre- and post-MSCT group.
