# Supplementary material for: Circulating miR-320b Contributes to CD4+ T-Cell Proliferation in Systemic Lupus Erythematosus via MAP3K1
Source: J Immunol Res. 2023 Oct 26;2023:6696967. doi: 10.1155/2023/6696967 (PMC10622187; doi:10.1155/2023/6696967)
Supplement: Supplementary 1 — Table S1: demographics and characteristics of SLE patients in miRNA sequencing. Table S2: demographics and characteristics of SLE patients for validation of miRNA candidates. Table S3: primers for reverse transcription and real-time polymerase chain reaction. Figure S1: the heatmap (a) and volcano plot (b) of differentially expressed miRNAs in SLE patients before and after receiving MSCT. Figure S2: the infection of PBMCs with shMAP3K1 lentivirus. [file 6696967.f1.docx]

**Supplementary Material**

**Table S1.** Demographics and characteristics of SLE patients in miRNA sequencing.

| **No.** | **Gender** | **Age (years)** | **Duration (months)** | **Baseline SLEDAI Score** | **Clinical manifestations** |
| --- | --- | --- | --- | --- | --- |
| **1** | F | 27 | 60 | 8 | LN, C, H, anti-dsDNA+, anti-SM+ |
| **2** | F | 58 | 38 | 17 | LN, C, F, P, ANA+ |
| **3** | F | 25 | 144 | 10 | LN, H, anti-dsDNA+, ANA+ |

ANA: antinuclear antibody; anti-dsDNA: anti double strand DNA antibody; C: cytopenia; F: febrile; H: hypocomplementemia; LN: lupus nephritis; P: polyserositis.

**Table S2.** Demographics and characteristics of SLE patients for validation of miRNA candidates.

| **No.** |  | **Gender** | **Age (years)** | **Baseline SLEDAI Score** | **Clinical manifestations** |
| --- | --- | --- | --- | --- | --- |
| **1** |  | F | 39 | 12 | LN, A, anti-dsDNA+, ANA+, anti-SM+ |
| **2** |  | F | 39 | 19 | LN, C, H, anti-dsDNA+, ANA+, anti-SM+ |
| **3** |  | F | 65 | 10 | LN, A, anti-dsDNA+, ANA+, anti-SM+ |
| **4** |  | F | 28 | 26 | LN, C, H, NPSLE, anti-dsDNA+ |
| **5** |  | F | 23 | 4 | LN, H, V, anti-dsDNA+, ANA+, anti-SM+ |
| **6** |  | F | 22 | 8 | LN, C, ANA+ |
| **7** |  | F | 30 | 8 | LN, anti-dsDNA+ |
| **8** |  | F | 35 | 8 | LN, C, H, P, ACA+, ANA+ |
| **9** |  | F | 22 | 23 | LN, C, H, P, anti-dsDNA+, ANA+ |
| **10** |  | M | 41 | 8 | LN, ANA+ |
| **11** |  | F | 30 | 17 | LN, C, H, ANA+ |
| **12** |  | F | 29 | 18 | LN, C, H, P, anti-dsDNA+, ANA+, anti-SM+ |
| **13** |  | F | 21 | 7 | LN, C, H, anti-dsDNA+, ANA+ |
| **14** |  | F | 48 | 8 | LN, A, anti-dsDNA+, ANA+ |
| **15** |  | F | 37 | 14 | LN, C, H, anti-dsDNA+, anti-SM+ |
| **16** |  | F | 44 | 6 | LN, C, H, ANA+, anti-SM+ |
| **17** |  | F | 31 | 7 | LN, C, H, anti-dsDNA+, ANA+ |
| **18** |  | M | 56 | 16 | LN, C, H, anti-dsDNA+ |
| **19** |  | F | 42 | 3 | C, H, anti-dsDNA+, ANA+, anti-SM+ |
| **20** |  | F | 14 | 16 | LN, C, H, anti-dsDNA+, ANA+, anti-SM+ |
| **21** |  | F | 18 | 10 | LN, C, F, H, anti-dsDNA+ |
| **22** |  | F | 24 | 5 | LN, C, anti-U1-RNP/Sm+ |
| **23** |  | F | 46 | 6 | LN, C, H, ANA+, anti-SM+ |
| **24** |  | F | 27 | 6 | LN, C, H, anti-dsDNA+, ANA+ |
| **25** |  | F | 35 | 15 | LN, C, anti-dsDNA+, ANA+, anti-SM+ |
| **26** |  | F | 27 | 8 | LN, C, H, anti-dsDNA+, anti-SM+ |
| **27** |  | F | 17 | 19 | LN, C, H, anti-dsDNA+, ANA+, anti-SM+ |
| **28** |  | F | 61 | 14 | LN, C, H, anti-dsDNA+, ANA+ |
| **29** |  | M | 26 | 17 | LN, C, H, V, anti-dsDNA+, ANA+ |
| **30** |  | F | 58 | 17 | LN, C, F, P, ANA+ |
| **31** |  | F | 32 | 18 | LN, H, ANA+, anti-SM+ |
| **32** |  | F | 31 | 16 | LN, H, ANA+ |
| **33** |  | F | 26 | 14 | LN, H, P, ANA+ |
| **34** |  | F | 34 | 8 | LN, H, anti-dsDNA+, ANA+ |
| **35** |  | F | 22 | 14 | LN, ANA+ |
| **36** |  | F | 35 | 16 | LN, H, ANA+ |
| **37** |  | F | 37 | 12 | LN, F, H, ANA+ |

A: arthralgia; ACA: anti-cardiolipin antibody; ANA: antinuclear antibody; anti-dsDNA: anti double strand DNA antibody; C: cytopenia; F: febrile; H: hypocomplementemia; LN: lupus nephritis; NPSLE: neuropsychiatric systemic lupus erythematosus; P: polyserositis; V: vasculitis.

**Table S3.** Primers for reverse transcription and real-time polymerase chain reaction.

| **Genes** | **Forward** | **Reverse** |
| --- | --- | --- |
| hsa-miR-320b | 5'-AAAAGCTGGGTTGAGAGGGCAA-3' |  |
| U6 | 5'-CGCTTCGGCAGCACATATACTA-3' | 5'-CGCTTCACGAATTTGCGTGTC-3' |
| 3’ primer | 5'-ATTCTAGAGGCCGAGGCGGCCGACATGT-3' |  |
| RT primer | 5'-ATTCTAGAGGCCGAGGCGGCCGACATGTTTTTTTTTTTTTTTTTTTTTTTTTTTTTT-3' | |
| hGAPDH | 5'-GCACCGTCAAGGCTGAGAAC-3' | 5'-TGGTGAAGACGCCAGTGGA-3' |
| hMAP3K1 | 5'-GTCACCACTCTTATTGTGCAGG-3' | 5'-TGGTTGTGAGTTGCACCAGA-3' |
| mGD | 5'-AGGTCGGTGTGAACGGATTTG-3' | 5'-TGTAGACCATGTAGTTGAGGTCA-3' |
| mMap3k1 | 5’-TACACTCCTTGCCACAGTCTGG-3' | 5'-CCTTGCAGAGTTCCAGCACTGT-3' |

**Fig. S1 A, B** The heatmap (A) and volcano plot (B) of differentially expressed miRNAs in SLE patients before and after receiving MSCT. MSCT: mesenchymal stem cell transplantation.

**Fig. S2** The infection of PBMCs with shMAP3K1 lentivirus. **A** The mRNA levels of MAP3K1 in PBMCs after lentivirus infection. **B** Representative images of GFP+ cells in PBMCs after lentivirus infection. PBMCs: peripheral blood mononuclear cell. n = 5. **p* < 0.05.
